# Integrated Transcriptome Analysis of Iris Tissues in Experimental Autoimmune Uveitis

**DOI:** 10.3389/fgene.2022.867492

**Published:** 2022-03-28

**Authors:** Dan Li, Chang Huang, Xiaoyan Han, Jianguo Sun

**Affiliations:** ^1^ Eye Institute, Eye & ENT Hospital of Fudan University, Shanghai, China; ^2^ Shanghai Key Laboratory of Visual Impairment and Restoration, Shanghai, China; ^3^ NHC Key Laboratory of Myopia, Fudan University, Shanghai, China; ^4^ Laboratory of Myopia, Chinese Academy of Medical Sciences, Shanghai, China

**Keywords:** autoimmune uveitis, iris, lncRNA, nod-like receptor (NLR), transcriptome analysis

## Abstract

Uveitis is a severe ocular inflammatory disease that affects the uvea and frequently results in visual impairment, even irreversible blindness. The current treatments for uveitis have exhibited adverse side effects. To find novel targets of this disease, we perform comparative transcriptome analysis using normal (*n* = 4) and experimental autoimmune uveitis (EAU) (*n* = 4) rat iris samples. We mainly focus on the expression profiles of mRNAs and long non-coding RNAs, and identify NOD-like receptor signaling pathway as the one that plays a key role in the pathological changes of the EAU irises. Our work demonstrates that the EAU iris transcriptome can be mined to uncover novel targetable pathways for uveitis. The molecules in NOD-like receptor signaling pathway could be novel therapeutic targets for autoimmune uveitis.

## Introduction

Uveitis is a common blinding eye disease that refers to inflammation of the iris, ciliary body, and choroid in the eyeball, which can cause or be accompanied by inflammation of all eye tissues. In severe cases, it can lead to the destruction of the patient’s ocular tissue structure and loss of function. It is one of the top three blinding factors in developed countries and China (Hou et al., 2015; Lee, 2015). In terms of etiology and types, there are nearly a hundred types of uveitis, which are mainly divided into two categories. One is infectious uveitis caused by bacteria, fungi, viruses, etc. through direct invasion of the uvea or retina; the other is non-infectious uveitis, also called autoimmune uveitis, caused by autoimmune reactions, most of which are accompanied by systemic diseases, such as Behcet disease, Vogt-Koyanagi Harada disease, rheumatoid arthritis, multiple sclerosis, etc. Judging from the number of clinical cases, we consider that non-infectious factors cause most uveitis.

Corticosteroids and immunomodulators are the primary drugs recommended to treat various forms of uveitis. Although these drug treatments are usually effective, they require long-term medication and may produce undesirable side effects. Among them, glucocorticoids are the most used drugs for the treatment of autoimmune uveitis, which have both anti-inflammatory and immunosuppressive effects. Long-term use of glucocorticoid eye drops is likely to cause increased intraocular pressure (glaucoma), posterior lens subcapsular opacity (cataract) and corneal epithelial damage; the side effects of long-term oral glucocorticoids include growth retardation in children, gastrointestinal perforation, muscle atrophy, and inducing tumor. Therefore, the development of new therapeutic targets for this disease is urgently needed.

The rodent model of experimental autoimmune uveitis (EAU) closely resembles the human condition and has been used to study the disease’s basic mechanisms. EAU can be induced in mice and rats by active immunization with retinal antigens such as interphotoreceptor retinoid-binding protein (IRBP) and retinal soluble antigen (S-Ag). The main features of EAU mice/rats include retinal and/or choroidal inflammation, photoreceptor destruction, and extensive inflammation in the eye (Chan et al., 1990; Chen et al., 2013). As a part of the uveal tract, the iris is the pigmented and muscular curtain of the eye, between the lens and the cornea. By controlling the size of the pupil, this tissue regulates the amount of light received by the special light-sensing cells in the retina. In a rodent EAU model, iris hyperemia is one of the most common inflammatory reactions. However, very few basic studies on non-infectious uveitis focus on the irises at the molecular level.

In the previous studies, the expression profiles have been characterized in retinas in the development of EAU, using gene microarray or RNA-seq analysis (Oh-i et al., 2007; Ahuja et al., 2008; Ishida et al., 2011; Watanabe et al., 2016; Lipski et al., 2017; Lipski et al., 2020). These studies have identified specific expression of protein-coding genes, such as alphaA-crystallin (Rao et al., 2008), genes for molecules associated with neuroprotection (Oh-i et al., 2007), and a series of microRNAs (Ishida et al., 2011; Watanabe et al., 2016). Furthermore, at the protein level, tandem mass tag peptide labeling coupled with LC-MS/MS quantitative proteomics technique has been applied. The differentially expressed proteins in EAU rat retinas were mainly associated with complement and coagulation cascades and metabolic pathways (Liu et al., 2020).

Different from previous studies, we collected iris samples from normal and EAU rats for whole transcriptome RNA-seq analysis. We identified the differentially expressed genes (DEGs), including mRNAs (DEmRNAs), lncRNAs (DElncRNAs), and microRNAs (DEmiRNAs) of iris samples from EAU and control rats. This study focused on the bioinformatic analysis of DEmRNAs and DElncRNAs. We found that the NOD-like receptor (NLR) receptor signaling pathway is the most enriched in the clustering analysis of DEGs, which is predicted to be the critical pathway in the pathological process in EAU.

## Methods and Materials

### Experimental Animals and Model Establishment

The animal study was reviewed and approved by the Ethics Committee of the Eye & ENT Hospital of Fudan University and abided by the Association for Research in Vision and Ophthalmology (ARVO) statement for the use of animals in ophthalmic and vision research.

Lewis rats were purchased from Shanghai Sippr-BK laboratory animal Co., Ltd., Shanghai, China. A total of 30 healthy male Lewis rats (180 ± 5 g) were fed at a temperature of 25°C and were exposed to light every 12 h. After 2 weeks of adaptive feeding, healthy Lewis rats were randomly divided into a normal control (NC) group (*n* = 15) and an EAU group (*n* = 15). To induce EAU, IRBP peptide R16 (1,177–1,191, ADGSSWEGVGVVPDV, 98% purity) emulsion was prepared:100 μg of R16 dissolved in 150 μL sterilized phosphate buffer saline (PBS). supplemented with 100 μg of *Mycobacterium* tuberculin H37RA (TB) and 150 μL of complete Freund’s adjuvant (CFA) to a total volume of 300 μL. Each rat in the EAU group was immunized subcutaneously with a total of 300 μL of IRBP R16 emulsion, while rats in the NC group received the identical volume containing only 150 μL of CFA plus 100 μg of TB (Yin et al., 2019).

### Ribonucleic Acid-Seq and Data Analysis

Total RNAs were extracted from iris samples from the EAU eyes (*n* = 4) and the NC eyes (*n* = 4) at day 14 after EAU induction, using TRIZOL reagent (Thermo Fisher Scientific, United States). The concentration and purity of the RNAs were checked using Nanodrop 2000 Spectrophotometer (Thermo Fisher Scientific). The RNAs’ integrity was checked using Agilent 2,100 Bioanalyzer (Agilent Technologies, United States). Ribosomal RNAs were removed from the total RNAs using TIANSeq rRNA Depletion Kit (TIANGEN Biotech, China) before the RNA library construction. The mRNA/lncRNA/circRNA sequencing library was prepared using TIANSeq Stranded RNA-Seq Kit (TIANGEN Biotech) for the Illumina platform, followed by RNA fragmentation, cDNA one-strand/two-strand synthesis, end repair, A-tail addition, adaptor ligation, cDNA two-strand digestion, and PCR enrichment. Paired-end reads (150 bp) were generated. The preprocessed data is aligned to the reference genome using HISAT (Kim et al., 2015). The miRNA sequencing library was prepared by the addition of adaptors to both ends of the miRNAs, reverse transcription to cDNAs, index addition, PCR amplification, and size selection. Using the program of cutadapt and fqtrim, the small RNA library adapter sequence contained in the read was removed, the base quality is less than 20 bases, and the read length greater than 18 nt is selected. The reads with the same sequence were further counted and merged into a unique sequence. Bowtie software (Langmead et al., 2009) was used to index the genome, and miRDeep software (Friedlander et al., 2008) was used to align the clean data to the reference genome. The reads aligned to the reference sequence were further aligned with the specified range sequences in mirbase (mirbase library version 22) to obtain the details of the matched miRNAs in each sample.

LncRNAs and mRNAs with the differential expression between the EAU and the control groups were identified by DESeq software (Robinson et al., 2010). The differentially expressed genes were identified with fold change criteria> 2; *p.* adjust <0.05. The mRNA/lncRNA raw sequences have been deposited in the NCBI database Sequence Read Archive (SRA) with the project number PRJNA810514.

### Functional Analysis of DEmRNAs and DElncRNAs

The enrichment analyses on DE gene sets were performed using the online tools: http://geneontology.org/ (for GO analysis), https://www.kegg.jp/ (for KEGG analysis). The protein-protein interaction networks were constructed using the online tool STRING: https://cn.string-db.org/ (for protein-protein interaction analysis). For statistical analysis, *p.* adjust <0.05 is considered significant. The bubble charts were drawn using R.

### Integrated Analysis of mRNAs-lncRNAs-miRNAs

We performed expression correlation analysis on lncRNA and mRNA, and calculated the correlation coefficient (Pearson correlation coefficient), *p* value and *p*. adjust value (adjusted by false discovery rate method, FDR) between any combination of lncRNA and mRNA, and then selected *p*. adjust less than 0.05 and the absolute value of the correlation coefficient greater than or equal to 0.99 as a reliable result. If the two are positively correlated, they are considered potential ceRNAs. Combined with the mRNA regulated by miRNAs predicted in the miRNA analysis results, miRNAs shared by lncRNAs and mRNAs can be found. Then, for the lncRNAs and mRNAs of shared miRNAs, if there is a positive correlation between the two, they are considered potential ceRNAs.

## Results

### Assessment of Experimental Autoimmune Uveitis Model

**FIGURE 1:**
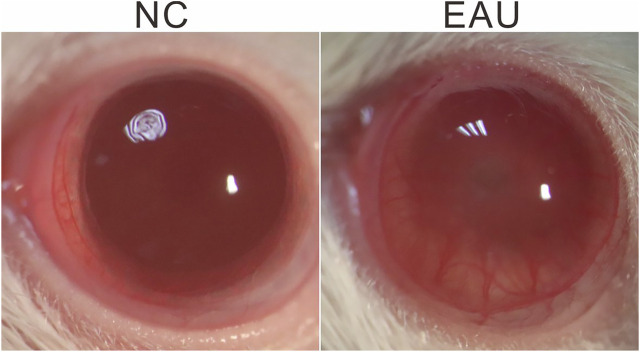
EAU phenotype. Representative images, taken on day 14 after EAU induction, demonstrate dilated iris blood vessels, hypopyon, severe miosis in an EAU eye. NC: normal control.

After the injection of IRBP R16 emulsion or NC solution (day 0), the severity of inflammation in EAU rat eyes was observed by microscopy examination of ocular inflammation, and the images were taken using a camera bound to the ocular lenses (Canon, Japan) on days 7 and 14. We observed severe iris hyperemia, hypopyon, and miosis in EAU eyes on day 14 (Figure 1), indicating a qualified model of EAU had been established.

### The Significant DEmRNAs and DElncRNAs in Experimental Autoimmune Uveitis Irises

**FIGURE 2:**
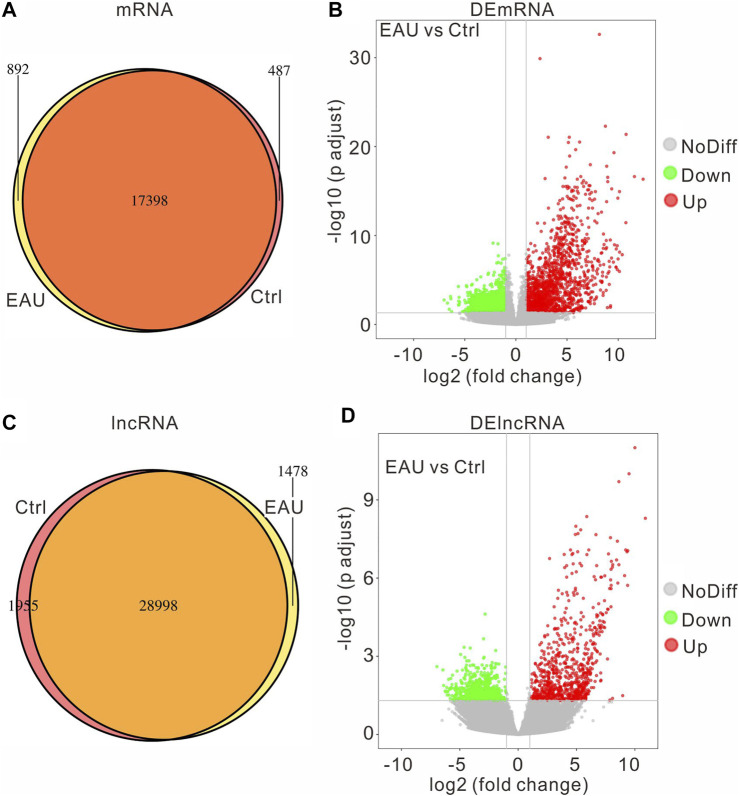
The expression profiles of mRNAs and lncRNAs. The amount of the common and the uniquely expressed mRNAs **(A)** and lncRNAs **(C)** are presented in Venn diagram. The volcano plot of differentially expressed mRNAs **(B)** and lncRNAs **(D)**.

To explore the molecular mechanisms involved in the EAU progress, we collected iris samples and performed an RNA sequencing assay to identify the DEGs in the irises. We compared the expressed genes of each group and got the following Venn diagram (Figures 2A,C): there are 892 uniquely-expressed mRNAs and 1,478 lncRNAs in the EAU group. The input data of DEG analysis is the read count data obtained in the analysis of gene expression level. The fold change >2, adjust *p* value <0.05 is considered significant through the statistical analysis. The volcano graph presents the DEmRNAs and DElncRNAs in the EAU irises (Figures 2B,D). There are 1710 upregulated and 1,646 downregulated DEmRNAs; 793 upregulated and 801 downregulated DElncRNAs. Notably, the change fold of the upregulated mRNAs and lncRNAs is higher than that of the downregulated ones.

### The Functional Analysis of DEmRNAs and DElncRNAs

**FIGURE 3:**
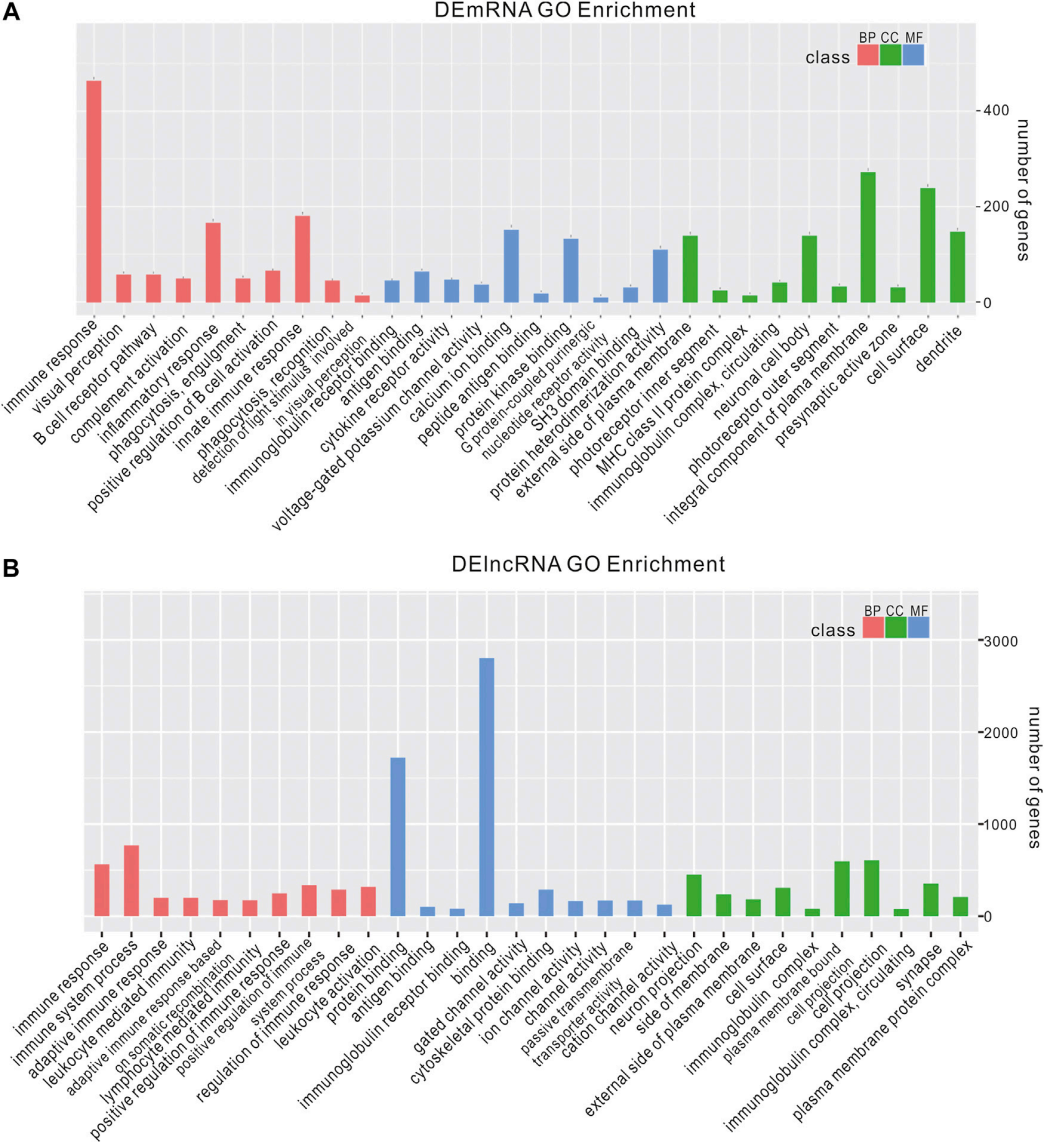
Enriched GO of DEmRNAs and DElncRNAs. top 10 most enriched GO for DEmRNAs **(A)** DElncRNAs **(B)** in EAU in terms of biological processes (BP), cellular component (CC), and molecular function (MF).

**FIGURE 4:**
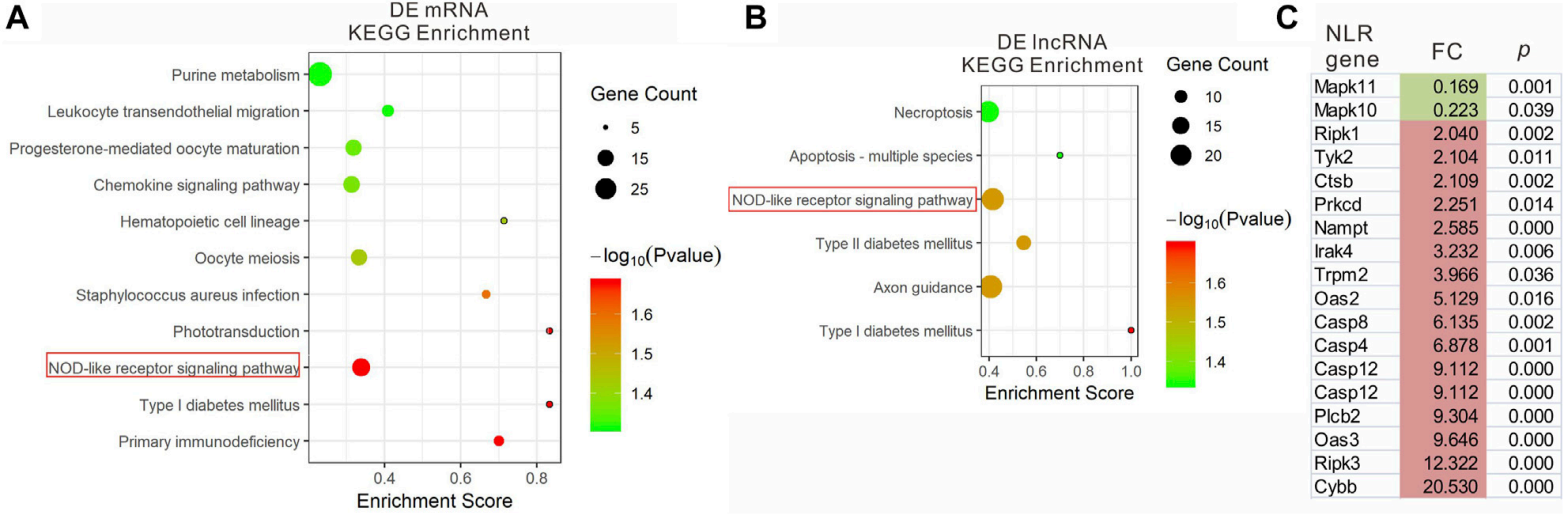
KEGG pathway enrichment analysis of DEmRNAs **(A)** and DElncRNAs **(B)** with top enrichment scores. **(C)** The fold change and *p*. adjust of the 18 DEGs in NRL signaling pathway.

We analyzed the functions of DEmRNAs via GO and KEGG pathway analyses. The GO enrichment includes Biological Process (BP), Molecular Function (MF) and Cellular Component (CC). The top 10 enriched GO terms in each category and enriched KEGG pathways (*p*.adjust <0.05) are presented (Figures 3A, 4A). The enriched GO and KEGG pathways of DElncRNAs were analyzed the same way as DEmRNAs, and the results are presented in Figures 3B, 4B. Notably, in the KEGG pathway analysis, the NLR-signaling pathway and Type I diabetes mellitus are the only two enriched pathways that exist in both the DEmRNAs and DElncRNA pathway analysis (Figures 4A,B). NLR signaling pathway has not been reported involved in the EAU development. In the NLR signaling pathway, 18 out of 53 genes were differentially expressed in the EAU group, which are *Tyk2*, *Casp4*, *Casp12*, *Prkcd*, *Ripk3*, *Casp1*, *Mapk10*, *Trpm2*, *Nampt*, *Irak4*, *Ripk1*, *Oas2*, *Oas3*, *Casp8*, *Ctsb*, *Cybb*, *Mapk11*, and *Plcb2*. The fold change and *p* value of these 18 genes is presented in Figure 4C. In the GO analysis of DEmRNAs, the enriched BPs concentrate on immune responses processes; the enriched CCs concentrate on the protein binding, including antigen binding, cytokine receptor binding, etc.; the enriched MFs focus on the cell surface, plasma membrane and photoreceptor. The results of GO analysis of DElncRNAs are quite similar with that of DEmRNAs, except that the enriched MFs include cell projection instead of photoreceptor (Figure 3).

**FIGURE 5:**
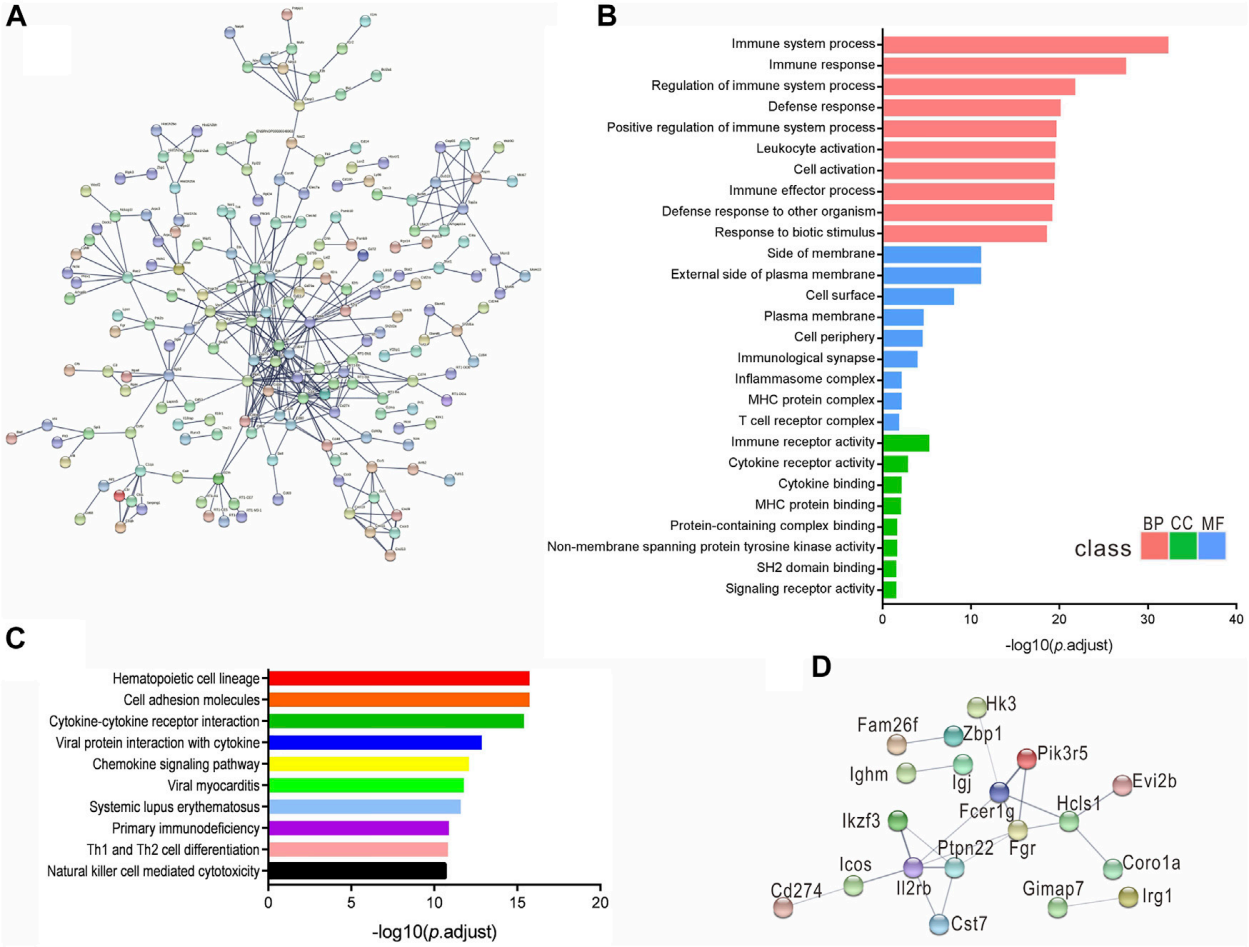
DEmRNAs protein-protein interaction networks. **(A)** Network established from 551 DEmRNAs with adjusted *p* value < 1 × 10^−5^, confidence score of 0.9. **(B,C)** Enriched GO and KEGG pathway of the interaction network. **(D)** The network constructed from the most significant 30 DEmRNAs, confidence score of 0.4.

We constructed a protein–protein interaction network of DEmRNAs using a set of 551 genes with the adjusted *p* < 1 × 10^–5^. The disconnected nodes were hidden to visualize the network better, and the highest confidence score of 0.9 was applied (Figure 5A). The GO and KEGG pathways identified from the network analysis are presented in Figures 5B,C. In addition, a concise network was established using a limited set of genes: the most significant 30 DEmRNAs, and the medium confidence score of 0.4 was applied (Figure 5D).

### Integrated Analysis of mRNAs-lncRNAs-miRNAs

Studies have recently discovered a wide-ranging regulatory interaction between various RNAs, including coding RNAs and non-coding RNAs (such as lncRNAs, pseudogenes, and circRNAs). These RNA transcripts compete with miRNAs through similar or identical target sites, thereby achieving mutual influence and regulation. This competitive mechanism is called competing endogenous RNAs (ceRNAs). This phenomenon reveals a mutual regulation between RNAs and provides a new direction for the study of the transcriptome.

**FIGURE 6:**
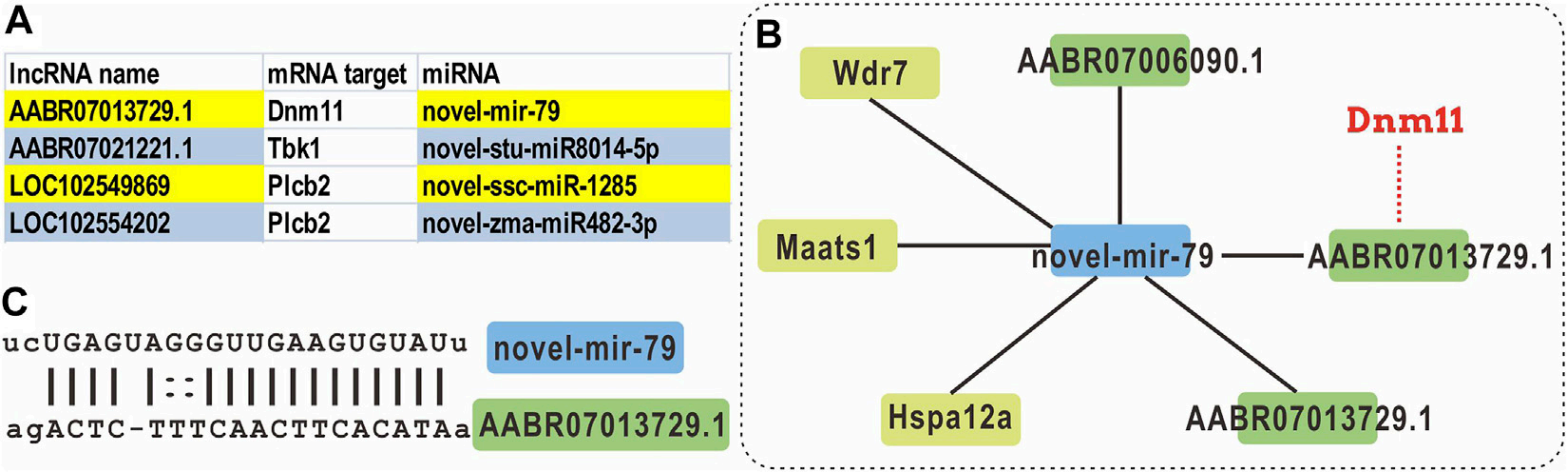
ceRNA analysis of the DElncRNAs with NLR signaling pathway gene targets. **(A)** The lncRNAs with an mRNA target that is categorized as a NRL signaling pathway gene, and its miRNA target. **(B)** The ceRNA network of novel-mir-79. **(C)** The alignment of novel-mir-79 and lncRNA AABR07013739.1.

Through the expression correlation analysis on lncRNA and mRNA, we identified the correlated expression patterns of mRNA-lncRNA. As presented in Table 1, four patterns were correlated counted: the relation between lncRNA and mRNAs are up-up, up-down, down-up, and down-down. Combining the miRNA expression profile and the predicted binding sites, we found 10,197 lncRNA-mRNA pairs share a miRNA sequence. We selected the DElncRNAs with a target mRNA gene in the NLR signaling pathway and constructed the ceRNA networks (Supplementary Figure S1 and Figure 6A).

**TABLE 1:** The expression correlation between DEmRNAs and DElncRNAs.

Group	LncRNA up-up mRNA	LncRNA up-down mRNA	LncRNA down- up mRNA	LncRNA down-down mRNA
EAU vs. Ctrl	5,569	24	103	107,659

For example, a novel miRNA novel-mir-79 is identified as the shared miRNA for three lncRNAs and three mRNAs (Figures 6B,C). Within this network, the mRNA target of lncRNA AABR07013729.1 is Dnm11, which is a component of NLR signaling pathway. However, there is no correlation when DEmRNAs, DElncRNAs, and DEmiRNAs are applied in the ceRNA analysis.

## Discussion

Autoimmune uveitis is an immune disease that affects the uvea, which comprises the iris, ciliary body, and choroid. Corticosteroids and other immunosuppressants are the golden standard in the management of the disease. However, chronic use of corticosteroids is associated with severe systemic side effects. Besides, depending on the severity of the inflammation, periocular or intraocular glucocorticoid injection is applied to reduce such morbidity. Its disadvantage is the complications caused by repeated injections, such as intraocular hemorrhage and proliferative vitreoretinopathy. To develop new therapies for this sight-threatening disease, biological treatments such as chimeric monoclonal antibody against TNF-α, or against adhesion molecules, complement components or other surface molecules involved in intercellular signaling or activation have been taken into research and clinical trials.

Toll-like receptors (TLRs) and NLRs are essential receptors that can both initiate innate immune responses and activate adaptive immune responses. These processes are bridges between innate immunity and adaptive immunity. In a study in yellow catfish, it is reported that under hypoxic stress, the NLR signaling pathway initially increased rapidly but then decreased over time, suggesting that the NLR-mediated immune response plays an essential role in hypoxia tolerance (Tao et al., 2021). In a study of choroidal neovascularization (CNV) using a laser-induced CNV mice model, it is reported that the DE transfer RNA-derived small RNAs (tsRNAs) are most enriched in NLR signaling pathway (Zhang et al., 2019). However, the NLR signaling pathway has not been reported as prominent in the studies of uveitis.

In our study, we identified DEmRNAs and DElncRNAs in EAU that are mostly enriched in the NLR signaling pathway. These NLR signaling enriched DEmRNAs encode three caspase genes: *Casp1*, *Casp4*, and *Casp12*. Mechanistically, NLRs exert their functions through three types of signal transduction pathways: the NF-κB pathway, the mitogen-activated protein kinase (MAPK) pathway, and the inflammasome pathway associated with IL-1β production. NLRP1, NLRP3, and NLRC4 induce caspase-1 activation through the assembly of inflammasomes. The activated caspase-1 regulates the maturation of the pro-inflammatory cytokines IL-1β, IL-18 and drives pyroptosis (Rathinam et al., 2012; Guo et al., 2015). Our data suggest a crucial role of the NLR signal transduction pathway in the EAU pathology in the iris region, which has not been identified as one of the most prominent pathways when using other uveal tissues, such as choroid/RPE samples. Notably, in a genetic association cohort study, exome sequencing analysis of the peripheral blood DNA samples of 164 patients with posterior segment uveitis identified *NOD2*, *NLRP1*, *NLRP3*, and *NLRC4* variants (Li et al., 2021). These findings add evidence to the conclusion that the NLR signaling pathway plays a key role in uveitis pathogenesis, and the molecules in this pathway could be novel therapeutic targets for autoimmune uveitis.

## Data Availability

The mRNA/lncRNA raw sequences have been deposited in the NCBI database Sequence Read Archive (SRA) with the project number PRJNA810514.
