# Supplementary figures and images for: Integrated Transcriptome Analysis of Iris Tissues in Experimental Autoimmune Uveitis
Source: Front Genet. 2022 Mar 28;13:867492. doi: 10.3389/fgene.2022.867492 (PMC8996140; doi:10.3389/fgene.2022.867492)

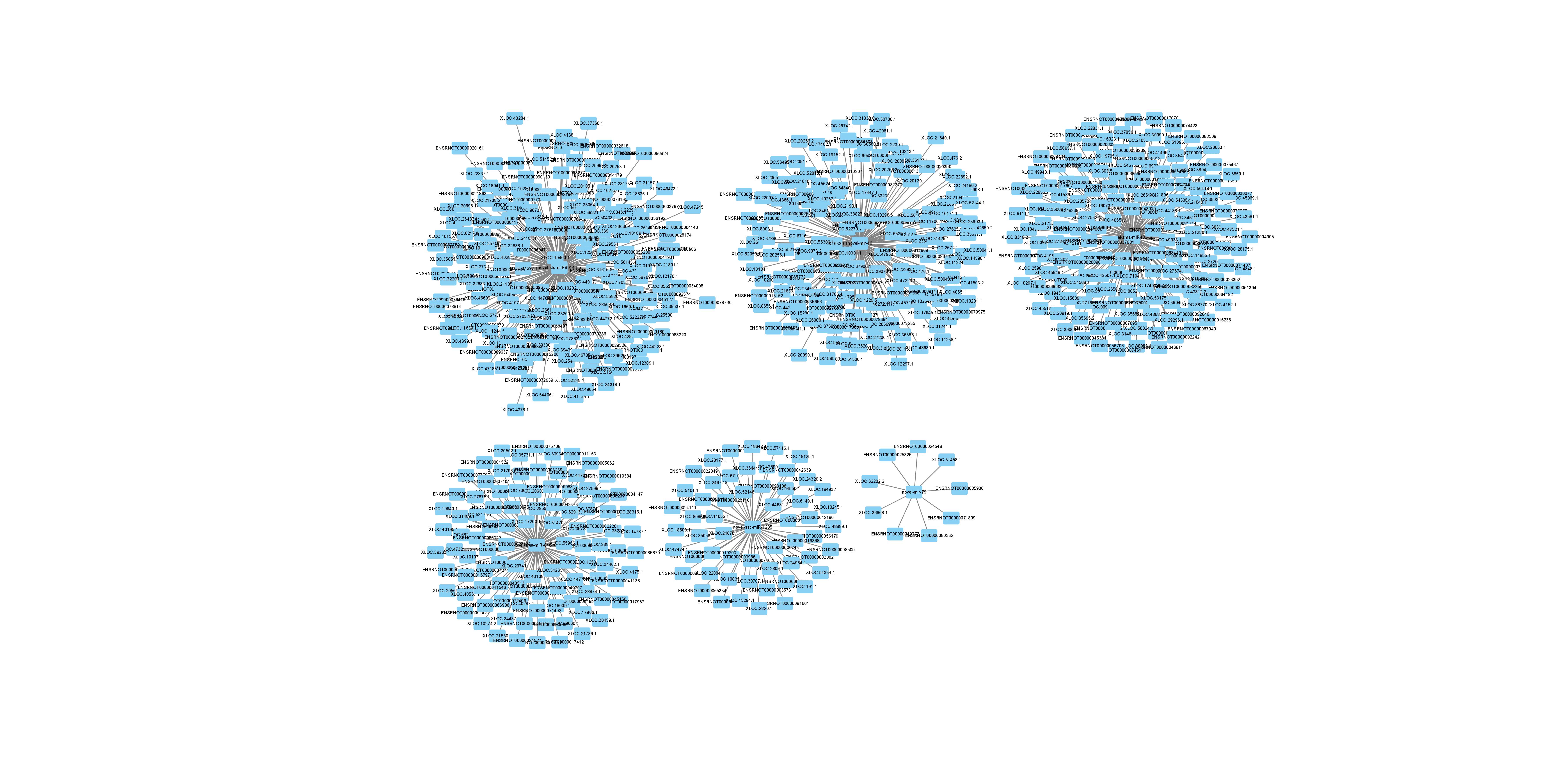

Supplement: Supplementary file 1 [file Image1.JPEG]
